# Social participation reduces depressive symptoms among older adults: An 18-year longitudinal analysis in Taiwan

**DOI:** 10.1186/1471-2458-11-292

**Published:** 2011-05-10

**Authors:** Chi Chiao, Li-Jen Weng, Amanda L Botticello

**Affiliations:** 1 Insitute of Health and Welfare Policy, Research Center for Health and Welfare Policy, School of Medicine, National Yang-Ming University, Taipei, Taiwan, China; 2 Department of Psychology, College of Science, National Taiwan University, Taipei, Taiwan, China; 3 Kessler Foundation Research Center & Department of Physical Medicine & Rehabilitation, UMDNJ-New Jersey Medical School, USA

**Keywords:** Depressive symptoms, Social participation, Older adults, Growth curve modeling, Taiwan

## Abstract

**Background:**

Relatively little empirical attention has focused on the association between social participation and depressive symptoms amongst older adults in Asian nations, where persons over the age of 65 represent a rapidly growing segment of the population. This study explores the dynamic relationship between participation in social activities and trajectories of depressive symptomatology among older Taiwanese adults surveyed over 18 years.

**Methods:**

Data are from a nationally representative sample of 1,388 adults aged 60-64 first surveyed in 1989 and followed over an 18-year time period for a total of six waves. Individual involvement in social activities was categorized into continuous participation, ceased participation before age 70, initiating participation in older adulthood, never participated, and dropped out before age 70. Two domains of depressive symptoms--negative affect and lack of positive affect--were measured using a 10-item version of the Center for Epidemiologic Studies-Depression Scale.

**Results:**

Analyses using growth curve modeling showed that continuously participating or initiating participation in social activities later life is significantly associated with fewer depressive symptoms among older Taiwanese adults, even after controlling for the confounding effects of aging, individual demographic differences, and health status.

**Conclusions:**

These findings suggest that maintaining or initiating social participation in later life benefits the mental health of older adults. Facilitating social activities among older adults is a promising direction for programs intended to promote mental health and successful aging among older adults in Taiwan.

## Background

Depression is one of the most common chronic mental health conditions among older adults in Chinese communities [1,2]. Symptoms of depression experienced in later life have serious implications for the health and functioning of older persons as emotional distress is consistently associated with higher levels of cognitive [3,4] and functional impairment [5,6], and the increased risk of physical illnesses such as heart disease and stroke. Depressive symptoms also place older adults at the increased risk for suicide [7-10], which can devastate families and communities.

Growing evidence suggests that involvement in social activities improves the mental health of older adults. Research has demonstrated that socially active older adults have better health outcomes than their inactive counterparts, such as lower mortality rates [11,12], better physical functioning [13], and higher cognitive functioning [14,15]. Work in this area suggests that participation in social activities provides older adults with social support from informal social networks (i.e., relationships with other social group members and peers), which in turn benefits their emotional functioning [16,17].

Prior studies examining the relationship between social participation and mental health also suggest that various forms of social participation have psychological advantages for older adults. For example, religious participation [18] and volunteer work [19] increased individual social resources (measured by meeting attendance and informal social interaction), which, in turn, lowered depressive symptom levels. Research by Li and Ferraro [20], Musick and Wilson (2003) [19], and Thoits and Hewitt (2001) [21] used longitudinal data from the Americans' Changing Lives study to explore the relationship between volunteering and depressive symptoms among older adults. These analyses each suggested that older adults psychologically benefited from sustained volunteering. Sugihara et al (2008) [22] examined the role of social participation in mitigating psychological distress in a nationally representative sample of Japanese adults aged 55 to 64. They found that volunteer work was significantly associated with fewer depressive symptoms for both males and females. Although these studies involved analysis of longitudinal data and identified a relationship between a particular type of social participation and mental health, one of the outstanding questions raised by prior investigations was how change and duration of social participation affected the development of psychological distress in later life. That is, variations in exposure to a protective resource--such as the continuity, initiation, or cessation of social participation--may differentially increase or decrease the likelihood of experiencing psychological distress among older adults [23].

This study seeks to increase our understanding of the complex association between social participation and depressive symptoms by analyzing this relationship from a lifecourse perspective. Our analysis uses a nationally representative sample of older adults from Taiwan, as Asian nations are less frequently the focus of empirical investigations of protective effects in mental health outcomes.

## Methods

### Sample and Data Collection

This study uses data collected from the Taiwan Longitudinal Study on Aging (TLSA), a nationally representative survey designed to study the impact of socioeconomic development on physical and emotional well-being of the older adult population in Taiwan. This prospective, longitudinal study involved data collection from 1989 to 2007 for a total of six waves. Detailed information on the study methodology and TLSA data collection is provided by the Bureau of Health Promotion at the Department of Health in Taiwan http://www.bhp.doh.gov.tw. The sample was derived with a multi-stage sampling framework. First, a total of 56 neighborhoods--defined as blocks or *lins*--were selected from nationwide administrative units. Second, individual persons aged 60 and older were selected within blocks, yielding the total original sample of N = 4,049. Participants were administered structured questionnaires in their homes by trained interviewers at baseline (1989), with follow-up surveys administered in 1993, 1996, 1999, 2003 and 2007. The response rates were 92%, 91%, 89%, 90%, 91% and 91% for each wave of data collection [24].

The analytic sample used for this study was restricted to those participants in the 60-64 year old age group at the baseline with complete data on the short form (10 items) of the Center of Epidemiological Studies-Depression scale from at least one follow-up. Our selection of this age group was based on three substantive considerations. First, this age group was not officially retired at baseline, which allowed us to examine changes in the relationship between participation and the distress both before and after retirement. Second, the life expectancy in 1989 in Taiwan was 71 years for males and 76 years for females [25]. As this study was based on the lifecourse perspective, we chose to examine the age group that was likely to survive for a large portion of the 18-year study. Thirdly, the age of 70 also provided a point of reference prior to the period of the lifecourse when we expected the risk for morbidity and mortality in our sample to increase. This selection yielded a final analytic sample of 1,388 older adults aged 60-64 years at the baseline, 1,174 in 1993, 1,047 in 1996, 960 in 1999, 800 in 2003, and 601 in 2007.

Study attrition over time was experienced in part due to longitudinal study design and the older adult sample. We assessed differences in social participation and individual characteristics between continuing participants and those participants that were lost to follow-up (the results not tabled). The analyses indicated that continuing participants at the sixth wave were significantly more likely to initiate participation in social activities in later life (OR = 1.43, *p *< 0.05) and had less physical limitations (OR = 0.61, *p *<  0.001) in comparison to the group that was lost to follow-up. The decline in sample size was primarily due to death.

### Measures

#### Dependent variable

Depressive symptomatology was measured by a 10-item version of the Center of Epidemiological Studies-Depression (CES-D) scale at each wave of the TLSA survey. The original 20-item CES-D [26] has been widely used in survey research to assess emotional distress in the general population, and has been demonstrated to have good validity and reliability when used with Asian populations [27-30]. Each of the 10 items was rated on a four-point scale (scored 0-3), indicating the frequency of experiencing each symptom in the past week. Responses were reversely scored when necessary such that higher scores represented greater levels of symptom frequency. Based on prior analyses using this sample [30-32], two factors were identified from the 10 CES-D items: a negative affect domain and a lack of positive affect domain. These CES-D items adopted in TLSA across waves are listed in the Appendix. More detailed information on psychometric properties of these two domains could be found in Chiao et al (2009) [29]. For the analysis, the items were summed within the two domains. The total score on the negative affect domain ranged from 0 to 24 with good internal consistency and reliability (α ranging from 0.79-0.87 across waves). The total score on the lack of positive affect domain ranged from 0 to 6 with an internal consistency reliability coefficient α of 0.79-0.95 across the waves.

#### Explanatory variable

Social participation was operationalized using items that measured social engagement. Participants reported whether they participated in group activities through any one of six types of social organizations: hobby-related clubs, religious or church groups, political groups, retired or elderly-related associations, or volunteer groups. For each type of social activity, older adults were further asked how long they had participated in an organization at each wave. As the objective of this study was to assess the potential dynamics between participation in social activities and distress during older adulthood over time, we used both participation in least one activity and participation duration to construct a measure of participation continuity by age of 70 using survey information from wave 1 to 3. The final social participation variable consists of the following five categories: (1) continuous social participation (from baseline to age 70); (2) ceased participation in older adulthood (between baseline and age 70); (3) initiating participation in older adulthood (after baseline); (4) never participated; and (5) dropped out before age 70.

#### Covariates

Age was included in all growth curve analyses as the time-varying covariate to assess change over time in depressive symptoms over the 18-year period. The relationship between aging and depressive symptoms has been reported as linear with a minor curvilinear effect [31]. Prior research has documented a robust association between physical limitations, chronic illness, and the mental health of older adults [33]. Therefore, indicators of physical health were assessed as covariates and measured by the presence or absence of *physical disability *and *chronic illness, respectively*. Physical disability was assessed from eight ADLs and IADLs items. The disability items assessed a person's difficulty with crouching, standing, stooping, lifting heavy objects, walking, climbing stairs, grasping small objects with their fingers, and taking a bus alone. We dichotomized disability status into those with no functional problems (coded "0") and those with at least one limitation (coded "1") following an approach used frequently in prior studies [34-37]. Chronic illness was assessed as a dichotomous measure (0 = no; 1 = yes) indicating whether respondents had medical diagnosis of at least one of the following five health problems: hypertension, diabetes, stroke, respiratory disease, and cardiovascular disease. Both disability and chronic illness status were based on health status reported at baseline.

The socio-demographic variables included *gender *and *ethnicity*. Ethnicity was categorized as Fukianese, Hakka, and Mainlander (i.e., individuals who fled the communist government of the People of Republic of China). Socioeconomic status (SES) was assessed by measures of *education*, *employment status*, and *home ownership*. The presence of family members in the immediate environment can be a source of both stress and social support for older adults [38,39] and is also an important feature of Taiwanese and Asian societies. Therefore, *family living arrangements *was included in the analysis and it was divided into two categories: living alone and living with extended family members. All sociodemographic indicators were based on measures obtained at baseline.

### Analytic Strategy

All analyses were conducted using STATA [40]. Sampling weights and statistical procedures with robust standard errors were also used throughout the analyses to correct for any potential biases. Bivariate tests (i.e., ANOVA and Bonferroni post hoc tests) were used to assess differences in the distribution of individual characteristics by types of social participation. Growth models calculated using the gllamm commands in STATA, were used to model depressive trajectories over the six waves of the study separately for the negative affect domain and the lack of positive affect domain. Two-level growth models were specified with individuals at level 2 and age at level 1 [41].

We used a sequential modeling strategy for the multivariate portion of the analysis, progressively adjusting our growth curve models to assess the relationship between social participation and change in depressive symptoms over time. The first model included individual social participation and age to examine whether there was significant variability in depressive symptoms over time for different categories of social participation. As suggested by prior research [31], the longitudinal relationship between aging and depressive symptoms is non-linear for Taiwanese older adults, and therefore we included a quadratic term in all growth curve models. The second model then added individual baseline controls (including health conditions, socio-demographic characteristics, and socioeconomic status) to the first model in order to assess the relative effect of social participation after adjusting for individual controls. As suggested by previous studies [22], an interaction term for individual social participation by gender was tested in all the models to explore any possible gender differences in depressive symptoms across the different participation categories.

## Results

Table 1 summarizes the distributions of individual characteristics stratified by categories of social participation. Approximately two-thirds of the sample reported no physical disability at baseline. Bivariate tests indicated that older adults who had better physical health status were more likely to be represented among the group that reported continuous social participation before age 70 (by wave 3) (71%) in contrast with 59% group who reported never participating and 57% of the group lost to follow-up. A relatively small portion of the sample (17%) reported experiencing chronic illness at baseline; unsurprisingly, persons with chronic illness were disproportionately represented among the group lost to follow-up midway through the TSLA data collection (i.e., by the age of 70). Due to a large number of male migrants from China in 1949, males comprised the majority of the sample (63.33%), and as expected, males and females exhibited significantly different patterns of participation in later life. We also observed significant differences in patterns of social participation by ethnicity, education level, work status, homeownership, and family living arrangement, as reported on Table 1. The continuous group reported lower levels of depressive symptoms on both domains in comparison to the never participated group and the dropped-out group.

**Table 1:** Sample characteristics [mean (SD) or percent] by categories of social participation at the 1989 baseline interviews in the Taiwan Longitudinal Study on Aging (TLSA)

	Continuous participation from baseline to age 70	Initiating participation from baseline to age 70	Ceased participation after baseline	Never participated before age 70	Dropped out before age 70	Total
	(n = 259)	(n = 274)	(n = 200)	(n = 359)	(n = 296)	(n = 1,388)
***Health status***						
Physical disability: One and more functioning limitations (%)	28.57^b^	33.94	33.50	40.67^a^	42.91^ab^**	36.53
Chronic disease: One and more chronic conditions (%)	15.83	13.50^a^	17.00	14.76	22.64^a^*	16.71
						
***Socio-demographic characteristics***						
Male (%)	80.31^aef^	57.66^ceg^	76.50^b^	43.73^abcd^	68.58^dfg^***	63.33
Ethnicity (%)						
Fukianese	51.35^a^	62.77^c^	47.50^bc^	65.74^ab^	58.11***	58.21
Hakka	15.06	16.06	14.00	15.32	10.14	14.12
Mainlander	33.59^ad^	21.17^def^	38.50^be^	18.94^abc^	31.76^cf^***	27.67
						
***Socioeconomic position***						
Education (%)						
Illiterate	17.76^ad^	31.02^d^	19.50^b^	38.44^abc^	25.68^c^***	27.67
Incomplete primary education	10.04	15.69	15.00	18.11	17.91	15.63
Completed primary education	36.68	33.58	34.00	27.02	28.72	31.48
High school graduate and above	35.52^ae^	19.71^cef^	31.50^bf^	16.43^abcd^	27.70^d^***	25.22
Work status (%)						
No work	27.03^b^	17.15^ac^	33.50	30.92^a^	40.54^bc^	29.90
Full- or part-time work	53.28^ac^	51.09^bd^	47.00	37.05^ab^	38.51^cd^***	44.60
Assisting family	19.69^ad^	31.75d^ef^	19.50^be^	32.03^abc^	20.95^cf^***	25.50
Home owned (%)	74.52^b^	78.10^d^	74.00^c^	72.70^a^	60.14^abcd^***	71.61
						
***Family background***						
Lives with family (%)	80.69^a^	81.75^b^	74.00	73.26	64.19^ab^***	74.50
						
***CES-D score***						
Negative affect domain	2.02^ad^ (2.94)	2.16^ce^ (3.22)	2.54^b^ (3.84)	3.55^abc^ (4.54)	3.52^de^*** (4.55)	2.84 (3.99)
Lack of positive affect domain	2.63^acd^ (2.35)	3.14^b^ (2.26)	3.51^c^ (2.32)	3.67^ab^ (2.14)	3.53^d^*** (2.18)	3.32 (2.27)

*p < 0.05; **p < 0.01; ***p < 0.001

^a, b, c, d, e, f, g ^Bonferroni post hoc tests: values with identical superscripts differ at the 0.05 level.

Table 2 presents the results of the growth curve analysis for the negative affect domain. Model 1 shows a significant effect of social participation on negative affect, independent of aging. In comparison to those who never participated in social activities, persons who continued or initiated their social participation as older adults have a significantly lower level of depressive symptoms (β_01_= -1.41, *p *< 0.001 and β_02_= -1.13, *p *< 0.001 respectively) over time. We also observed a modest negative association between participation and negative affect over time (β_03_= -0.71, *p *< 0.05) for those who ceased participation before age 70, suggesting that even unsustained social activity (as compared to no involvement) in older adulthood is psychologically beneficial in the long term. As for the aging effect, levels of depressive symptoms on the negative affect domain increased with age (mean linear growth rate = 0.18, *p *< 0.001) with the acceleration in the likelihood of symptoms diminishing slightly over time (mean quadric growth rate = -0.004, *p *< 0.01).

**Table 2:** Growth curve models predicting depressive symptomatology on the *negative affect *domain, the Taiwan Longitudinal Study on Aging (TLSA) 1989-2007

	Model 1		Model 2	
	
*Covariates*	Coefficient (β)	Standard Error	Coefficient (β)	Standard Error
***Social participation***				
Participation before 70 (ref = never participated)				
Continuous participation from baseline to age 70, β_01_	-1.414***	0.262	-0.681**	0.250
Initiating participation from baseline to age 70, β_02_	-1.128***	0.256	-0.709**	0.235
Ceased participation after baseline, β_03_	-0.710***	0.283	-0.195**	0.265
Dropped out before age 70, β_04_	0.003**	0.277	0.111**	0.262
				
***Health status***				
Physical disability (ref = None)				
One and more functioning limitations			1.789***	0.180
Chronic health problems (ref = None)				
One and more chronic conditions			-0.297***	0.221
***Socio-demographics***				
Gender (ref = Male)				
Female			0.294***	0.221
Ethnicity (ref = Fukianese)				
Hakka			0.017***	0.240
Mainlander			-0.122***	0.218
***Socioeconomic position***				
Education (ref = Illiterate)				
Incomplete primary education			-0.434***	0.272
Completed primary education			-0.941***	0.226
High school graduate and above			-1.437***	0.265
Work status (ref = No work)				
Full- or part-time work			-0.690***	0.201
Assisting family			-0.754***	0.238
Home ownership (ref = no)			-0.465***	0.188
***Family background***				
Family living arrangement (ref = Alone)				
Living with family			-0.534***	0.200
				
Symptom level, intercept	3.142***	0.209	4.146***	0.367
Mean growth rate, linear	0.182**	0.031	0.175***	0.030
Mean acceleration, quadratic	-0.004***	0.001	-0.004**	0.001

*Random effects*	Estimate	S.E.	Estimate	S.E.

Variance in random intercept	4.285***	0.735	2.392***	0.599
Variance in random slope	0.018***	0.006	0.017***	0.005
Variance in residuals	13.297***	0.342	13.335***	0.318

*p < 0.05; **p < 0.01; ***p < 0.001

In order to assess the effects of confounding factors, the individual covariates were included in subsequent models. Although though the coefficients for those who continued or initiated their social participation remain significant, the magnitude of these associations was appreciably reduced after adjusting for individual differences in health status (i.e., presence of disability or chronic illness) socio-demographic characteristics, and socioeconomic status. The observed relationship between participation and negative affect for the group who ceased participation was rendered non-significant when health and socio-demographic differences were taken into account. Experiencing physical disability increased the level of negative affect over time. Conversely, the level of negative affect decreased with higher education attainment, with full- or part-time employment (versus not employed), with home ownership (versus not), and with living with family members (versus living alone). Contingencies between gender and social participation were found to be non-significant (model not tabled), suggesting that the protective effect of social participation on negative affect did not differ between men and women.

Table 3 presents the results for the lack of positive affect domain. Model 1 showed that the likelihood for a lack of positive affect was decreased by either continued or initiated social participation in older adulthood (β_01_= -0.74, *p *< 0.001 and β_02_= -0.48, *p *< 0.001 respectively), as compared to never participating. We also observed a modest negative relationship among those individuals who ceased participation in older adulthood; as before, this suggests that some participation in social activities in later life is better than nothing at all. The overall relationship between social participation and lack of positive affect persisted even after adjusting for individual differences (Model 2). As with the negative affect domain, presence of a disability increased the tendency to experience a lack of positive affect over time and slightly attenuated some of the psychological benefit of participation on long-term depressive symptomatology. Our results demonstrated that lack of positive affect decreased with age, suggesting that this aspect of depressive symptomatology changes, at least in part, with the passage of time.

**Table 3:** Growth curve models predicting depressive symptomatology on the *lack of positive affect *domain, the Taiwan Longitudinal Study on Aging (TLSA) 1989-2007

	Model 1		Model 2	
*Covariates*	Coefficient (β)	Standard Error	Coefficient (β)	Standard Error
***Social participation***				
Participation before 70 (ref = never participated)				
Continuous participation from baseline to age 70, β_01_	-0.736***	0.107	-0.600***	0.108
Initiating participation from baseline to age 70, β_02_	-0.472***	0.104	-0.398***	0.102
Ceased participation after baseline, β_03_	-0.203***	0.115	-0.114***	0.115
Dropped out before age 70, β_04_	-0.092***	0.124	-0.073***	0.123
***Health status***				
Physical disability (ref = None)				
1+ functioning limitations			0.321***	0.080
Chronic health problems (ref = None)				
1+ chronic conditions			-0.128***	0.099
***Socio-demographics***				
Gender (ref = Male)				
Female			-0.250***	0.098
Ethnicity (ref = Fukianese)				
Hakka			-0.053***	0.106
Mainlander			+0.012***	0.097
***Socioeconomic position***				
Education (ref = Illiterate)				
Incomplete primary education			-0.351***	0.120
Completed primary education			-0.502***	0.099
High school graduate and above			-0.697***	0.116
Work status (ref = No work)				
Full- or part-time work			-0.117***	0.189
Assisting family			-0.009***	0.156
Home ownership (ref = no)			-0.132***	0.083
***Family background***				
Family living arrangement (ref = Alone)				
Living with family			-0.185***	0.088
				
Symptom level, intercept	3.814***	0.103	4.443***	0.171
Mean growth rate, linear	-0.172***	0.018	-0.173***	0.018
Mean acceleration, quadratic	0.005***	0.001	0.005***	0.001

*Random effects*	Estimate	S.E.	Estimate	S.E.

Variance in random intercept	0.952***	0.196	0.874**	0.192
Variance in random slope	0.002***	0.001	0.002**	0.001
Variance in residuals	4.557***	0.106	4.546***	0.106

*p < 0.05; **p < 0.01; ***p < 0.001

## Discussion

This study suggests that overall, social participation benefits the mental health of older Taiwanese adults. Our analysis also illustrates the dynamic nature of this relationship; that is, our results demonstrate that social involvement that is continued or initiated in later life is protective of mental health over and above individual differences in social circumstances and health status. However, our observation of a modest effect for those adults who had to cease social participation in their 70's also suggested that some involvement in social activities in later life is better for mental health as compared to no social participation at all. This observed association was accounted for by individual differences - specifically, physical disability - suggesting that functional impairments are a major threat to social activity later in life.

This study adds to the body of research showing the benefits of remaining socially active in later life, as demonstrated by lower depressive symptom levels for adults who reported continued involvement in social activities versus adults who reported no social participation. As suggested by our analyses, making a continuous effort to participate in social activities in late life is a commitment to preserving the older person's mental health, even though such participation may be varied by different types of social activities and it may also be caused by many other potentially motivating factors such as the desire to attend social functions and a search for emotional support. Our analysis also demonstrates that it is never too late to reap the psychological benefits of human interaction.

Previous studies have suggested a gender difference in the relationship between social participation and mental health status among older adults [22]. However, our analyses did not yield any gender differences in both domains of depressive symptoms. This discrepancy may be due to the relatively small female subgroup (N = 51) that reported continuous social participation over the course of the study. In other words, the statistical power for detecting this particular relationship might be quite low. More work is needed to assess the potential gender differences in social participation and the effect of this dynamic variable on psychological distress among Taiwanese older adults [42].

Although our work provides important insights into the social aspects of mental health for older adults in non-Western countries using longitudinal data, we acknowledge that our approach has several limitations. First, the scope of this study was limited to social participation in general. As the Taiwanese place a high value on family, future research needs to specifically compare the influence of social participation through family versus community activities. Second, in order to maximize our analytic sample by minimizing the risk of the attrition over time, we focused on a sample aged from 60 to 64 years with 18 years of follow-up data, a relatively "young" and healthy group of older adults. This selected sample limits the generalizability of the results to the "youngest old," who are likely to have higher rates of social participation than members of the older cohorts due to better health, less disability, and wider social circles. Third, the TLSA data is based on self-reported recall of social activities and depressive symptoms, raising the issues of recall bias. Fourth, the individual controls used in the analyses are based on baseline measures. Several of these variables such as health status and family living arrangement were likely to have changed over the 18-year period of study. Analysis of the additional time-varying covariates was beyond the scope of this investigation which focused on the time-varying nature of social participation and the relationship of this focal construct with mental health. The next logical step in this line of inquiry is to investigate the role of changes in social support, health and disability status experienced by older adults in the pathway between social participation and emotional health.

## Conclusions

This study extends prior research concerning the longitudinal relationship between aging and depressive symptomatology to address gaps in the empirical literature on the association between social participation and distress among older members of the Taiwanese population. Our analyses demonstrated that social participation is globally beneficial to the psychological health of older adults and that specifically, continued or initiated social activities mitigates depressive symptoms that are likely to be experienced in later life. This study also contributes to the growing interest in late-life social participation and mental health for a population that is not frequently the focus of empirical studies in mental health and aging. Public policy and healthcare interventions aiming at promoting social participation for older adults represent a promising area for maintaining good mental health among a growing segment of Taiwanese society.

## Note

TLSA data are openly available and can be applied for research use by approval of the Bureau of Health Promotion at the Department of Health in Taiwan.

## Appendix: The 10 CES-D items used across the waves in TLSA

Everyone has mood changes. In the past week, have you experienced the following situations or feelings? If yes, continue to ask: Does this happen to you rarely (one day in the past week), sometimes (2-3 days in the past week), often or chronically (over 4 days in the past week)?

### Negative affect

1. Not interested in eating, have a poor appetite.

2. Feel that doing everything was exhausting.

3. Sleep poorly (unable to sleep soundly).

4. Feel you were in a terrible mood.

5. Feel lonely (isolated, with no companion).

6. Feel people around you weren't nice to you (unfriendly).

7. Feel anguish.

8. Unable to gather your energy to do things (Had no will to do anything).

### Lack of positive affect: reverse scored

1. Feel joyful.

2. Feel that your life was going well.

## Competing interests

The authors declare that they have no competing interests.

## Authors' contributions

CC was responsible for development of study hypotheses, data analysis, and drafting of the article. LJW contributed to developing study hypotheses and critical revision of the article. ALB contributed to critical revision of the article. All authors involved in the writing of the paper, and all approved the final submission.

## Pre-publication history

The pre-publication history for this paper can be accessed here:

http://www.biomedcentral.com/1471-2458/11/292/prepub
